# Lessons learned for surveillance strategies for trachoma elimination as a public health problem, from the evaluation of approaches utilised by Guinea worm and onchocerciasis programmes: A literature review

**DOI:** 10.1371/journal.pntd.0009082

**Published:** 2021-01-28

**Authors:** Laura Senyonjo, Philip Downs, Elena Schmidt, Robin Bailey, Karl Blanchet

**Affiliations:** 1 Research Team, Sightsavers, Haywards Heath, United Kingdom; 2 Clinical Research Department, London School of Hygiene & Tropical Medicine, London, United Kingdom; 3 Neglected Tropical Diseases Department, Sightsavers, Haywards Heath, United Kingdom; 4 Department of Global Health and Development, London School of Hygiene & Tropical Medicine, London, United Kingdom; Emory University, UNITED STATES

## Abstract

**Introduction:**

A number of neglected tropical diseases are targeted for elimination or eradication. An effective surveillance system is critical to determine if these goals have been achieved and maintained. Trachoma has two related but morphologically different presentations that are monitored for elimination, the active infectious form of trachoma and trachomatous trichiasis (TT), the progression of the disease. There are a number of lessons learnt from the Guinea worm surveillance system that are particularly compatible for TT surveillance and the onchocerciasis surveillance system which can provide insights for surveillance of the infectious form of trachoma.

**Methods/Principal findings:**

A literature search of peer-reviewed published papers and grey literature was conducted using PUBMED and Google Scholar for articles relating to dracunculiasis or Guinea worm, onchocerciasis and trachoma, along with surveillance or elimination or eradication. The abstracts of relevant papers were read and inclusion was determined based on specified inclusion and exclusion criteria. The credibility and bias of relevant papers were also critically assessed using published criteria. A total of 41 papers were identified that were eligible for inclusion into the review.

The Guinea worm programme is designed around a surveillance-containment strategy and combines both active and passive surveillance approaches, with a focus on village-based surveillance and reporting. Although rumour reporting and a monetary incentive for the identification of confirmed Guinea worm cases have been reported as successful for identifying previously unknown transmission there is little unbiased evidence to support this conclusion. More rigorous evidence through a randomised controlled trial, influenced by motivational factors identified through formative research, would be necessary in order to consider applicability for TT case finding in an elimination setting. The onchocerciasis surveillance strategy focuses on active surveillance through sentinel surveillance of villages and breeding sites. It relies on an entomological component, monitoring infectivity rates of black flies and an epidemiological component, tracking exposure to infection in humans. Challenges have included the introduction of relatively complex diagnostics that are not readily available in onchocerciasis endemic countries and target thresholds, which are practically unattainable with current diagnostic tests. Although there is utility in monitoring for infection and serological markers in trachoma surveillance, it is important that adequate considerations are made to ensure evidence-based and achievable guidelines for their utility are put in place.

**Conclusions/Significance:**

The experiences of both the Guinea worm and onchocerciasis surveillance strategies have very useful lessons for trachoma surveillance, pre- and post-validation. The use of a monetary reward for identification of TT cases and further exploration into the use of infection and serological indicators particularly in a post-validation setting to assist in identifying recrudescence would be of particular relevance. The next step would be a real-world evaluation of their relative applicability for trachoma surveillance.

## Author summary

The design of a surveillance system needs to be carefully thought out to ensure it provides sufficient evidence to determine if a disease or infection is eliminated or eradicated. If inappropriate it can lead to on-going transmission and resurgence of infection or disease or the unnecessary continuation of interventions, wasting valuable resources. Guinea worm is a disease that is painful and debilitating, for which there is no drug or vaccine. The aim is to eradicate the disease and as such the Guinea worm programme is designed around a strategy of identification of cases and their containment to prevent onward transmission. Onchocerciasis if left untreated can lead to blindness. The aim is to eliminate the disease through the interruption of transmission. A literature review was conducted to determine available evidence and identify lessons that can be learnt from the surveillance of both diseases for the design of trachoma surveillance strategies in the endgame. The potential utility of rumour reporting and a monetary incentive for the identification of a confirmed case of Guinea worm could be explored for trichiasis case finding. Trichiasis is the progression of trachoma and leads to significant ocular morbidity. The introduction of tests for infection and antibodies and the utility of sentinel surveillance as utilised for onchocerciasis are interesting considerations for active trachoma surveillance post-validation and has potential to identify recrudescence cost-effectively. The experiences of both the Guinea worm and onchocerciasis surveillance strategies have very useful lessons that can be trialled for trachoma surveillance. However, their real-world applicability and implications for trachoma need to be evaluated before any changes in guidelines are proposed.

## Introduction

The 2012 World Health Organisation (WHO) roadmap on neglected tropical diseases (NTDs) outlines an ambitious plan for the control, elimination and eradication of at least 17 diseases [1], further endorsed by the London Declaration on NTDs [2]. Three of these NTDs include dracunculiasis, commonly known as Guinea worm, which along with yaws is one of two NTDs targeted for eradication; onchocerciasis targeted for elimination (interruption of transmission) in the majority of African countries and trachoma targeted for elimination as a public health problem, all by 2030 [3]. Determining whether a disease is suitable for eradication or elimination takes into account a number of factors, including transmission dynamics, availability and performance of diagnostic tests and interventions [4,5].

The WHO defines eradication as “the permanent reduction to zero of the worldwide incidence of infection caused by a specific pathogen established in a human or animal population, as a result of deliberate efforts, with no more risk of reintroduction” [6]. To date eradication has only been achieved in the case of smallpox [7] and rinderpest [8]. Once eradication is achieved worldwide, routine interventions and surveillance can be stopped. Elimination refers to the reduction in infection or transmission to zero or to the degree that interventions can stop in a defined geographical area, although post-elimination surveillance must continue [9,10].

For each disease a specific surveillance system has been designed in order to provide the required evidence that elimination or eradication targets have been achieved and interventions can stop. The sensitivity of such a system is important in order to ensure that elimination or eradication thresholds have been achieved and interventions are not stopped prematurely, risking recrudescence. In an eradication programme or where the goal is interruption of transmission, it is vital to have extremely sensitive surveillance measures to provide confidence that all cases are detected [11]. Specificity of a surveillance system is also important in order to limit the proportion of false positives and ensure that interventions do not continue unnecessarily, or that declaration of elimination or eradication is not delayed, both scenarios potentially wasting limited resources [12].

This paper will review the structure and performance of the various surveillance strategies utilised by Guinea worm eradication and onchocerciasis elimination programmes and discuss the utility of the approaches for trachoma surveillance. NTD monitoring can be categorised into four phases, mapping to establish disease prevalence at baseline, progress or impact monitoring after interventions have started, evaluation of stopping decisions to determine if elimination or eradication thresholds have been reached and post-intervention or elimination surveillance to monitor for recrudescence [12]. This paper will only focus on surveillance related to the latter two phases. Information on the three diseases, their transmission and recommended interventions are summarised in Table 1.

**Table 1:** A summary of the disease profiles and current interventions for trachoma, Guinea worm and onchocerciasis.

	Trachoma	Guinea worm	Onchocerciasis
Causative agent	*Chlamydia trachomatis* (*Ct*) (bacterium)	*Dracunculus medinensis* (parasitic worm)	*Onchocerca volvulus* (filarial nematode)
Significant morbidity	Trachomatous trichiasis (TT), the in-turning of the eyelashes so they touch the globe of the eye, scratching the cornea and leading directly or indirectly to corneal opacity	Slow and painful emergence of the female nematode, usually on the lower limbs	Inflammation in the eye can lead to significant ocular morbidity or blindness
Primary transmission route	Direct contact through fomites and fingers or eye-seeking flies [13].	Drinking stagnant water contaminated with copepods containing the infective *D*. *medinensis* larvae. An unusual epidemiology in Chad, with dogs infected with Guinea worm genetically indistinguishable from *D*. *medinensis* and simultaneous sporadic unlinked infections in humans, suggested an alternative transmission cycle and the potential of a paratenic or transport host [14]	Bite of an infected blackfly of the genus *Simulium*, which breed in fast-flowing water [15]
Reservoir of infection	Humans	Primarily humans but infections also identified in dogs [14], baboons and cats [16]	Humans
Key interventions for elimination or eradication efforts	WHO-endorsed SAFE strategy (S—surgical intervention for trichiasis; A—antibiotics to treat active infection, F—facial cleanliness and E–environmental improvements) [17]	No known anti-helminthic medication or vaccine. Reliance on the rapid identification and containment of cases, effective behaviour change strategies, improved potable water supply and vector control through the treatment of infected water using an effective larvicide [18]	Long-term annual or semi-annual mass treatment with ivermectin, a microfilaricide [19]
Current progress towards elimination or eradication	As of September 2020, ten countries have been validation as eliminated trachoma as a public health problem, seven in Asia (Cambodia, China, Islamic Republic of Iran, Lao People’s Democratic Republic, Myanmar, Nepal and Oman), one in South America (Mexico) and two in Africa (Ghana and Morocco) [20,21]	There has been huge progress towards Guinea worm eradication, with 3.5 million cases reported in the 1980s, down to only 28 cases by the end of 2018 [6]. However, the endgame has taken longer than expected and challenges remain. In 2018, the first ever case of Guinea worm was identified in Angola, approximately 2000 kilometres away from any known endemic area [6]	Onchocerciasis elimination has made great strides in the Americas and although onchocerciasis blindness as a result of infection is no longer a public health problem across the majority of Africa, the goal of elimination and interruption of transmission has had more limited successes [22]

As country programmes progress along the trachoma elimination pathway, it is imperative that evidence-based guidance is available, summarising the most effective surveillance strategies to identify recrudescence of infection or disease. Guidance for pre-validation surveillance has in part been guided by expert opinion due to a lack of available evidence in some areas, such as the shift in strategy from continuous active surveillance of trachomatous—inflammation follicular (TF) in sentinel communities (post-MDA stopping) [23] to the repeat of a population-based survey only [24]. There are currently no formal guidelines on post-validation trachoma surveillance and a paucity of information on risks of recrudescence, hampering their development. Where evidence is lacking it is possible to learn lessons in regards to strategies from other disease surveillance programmes. The clinical progression of trachoma with the active infectious form of trachoma and TT, the progression of the disease, two related but morphologically different presentations monitored for elimination, adds complexity to trachoma surveillance, which require alternative surveillance strategies. There are a number of lessons learnt from the Guinea worm surveillance system that are particularly compatible for TT surveillance while the surveillance of onchocerciasis can provide insights for surveillance of the active infectious form of trachoma.

## Methodology

A literature search of peer-reviewed published papers and grey literature was conducted using PUBMED and Google Scholar. The search strategy used search terms specific to each disease, ‘dracunculiasis’ or ‘Guinea worm’, ‘onchocerciasis’ and ‘trachoma’ in combination with ‘surveillance’ or ‘elimination’ or ‘eradication’. A more general search for ‘neglected tropical disease’ and ‘surveillance’ was also included. All search terms and their variations are outlined in the supplementary information (S1 Table). The search was conducted in July 2019, there was no retrospective date restriction to the sources identified.

The abstracts of papers retrieved following the search were read and reviewed against a set of inclusion criteria.

### Inclusion criteria

Papers were included in the review, if they

- referred to one of the diseases under study (Guinea worm, onchocerciasis or trachoma);
- described international or national frameworks or surveillance guidelines;
- presented experiences in using national or local surveillance strategies for elimination or for a stop-intervention decision;
- presented primary research or data related to an evaluation of the performance of the surveillance system;
- available in the English language.

#### Exclusion criteria

Papers were excluded from the review, if they

- were purely descriptive progress reports on case numbers or findings of impact assessments for routine or WHO recommended approaches;
- evaluated or compared the performance of specific diagnostic tests;
- referred to baseline mapping or pre-control surveillance only;
- referred to developed countries only.

Where articles presented evaluation findings (qualitative or a randomised controlled trial (RCT)), these were critically reviewed for quality and strength of evidence using the appropriate critical appraisal skills programme checklist [25]. Surveys were reviewed using the Center for Evidence-based Management critical appraisal of a survey tool [26]. Based on the assessment, papers were credited as one of three categories, i) likely credible or low risk of bias, ii) unclear credibility or risk of bias and iii) unlikely credible or high risk of bias. Guidelines and papers outlining country experiences were excluded from the critical appraisal.

### Findings

A total of 41 papers met inclusion criteria and were included in the review, 14 relating to Guinea worm, 8 relating to onchocerciasis, 18 relating to trachoma and one relating to more than one of these diseases. Of these, 17 were primary studies or evaluations relating to surveillance performance, 12 were descriptions of country experiences, eight presented guidelines or frameworks for elimination surveillance, two were modelling papers and two were reviews of applicability of current guidelines. Of the primary studies or evaluations, two were qualitative studies, three used mixed methods, 10 were cross-sectional surveys, and two were RCTs, all 17 of which were critically appraised. Out of these, 13 were deemed as credible and a low risk of bias, one had low credibility and high risk of bias and for three there was unclear credibility and risk of bias. The main reasons for low or unclear credibility were the lack of information on methodology or tools and limited consideration of potential sources of bias. The papers included in the review are outlined in the supplementary information (S2 Table).

i Overview of surveillance systems

The three diseases included in this review have different global targets, which influence the design of the surveillance strategies. Guinea worm is targeted for eradication, with surveillance designed around a case-based surveillance-containment strategy. It is imperative that cases are quickly identified (ideally within 24 hours), confirmed and contained before onward transmission [27]. The Guinea worm programme combines the use of both active and passive surveillance approaches, with a focus on village-based surveillance and reporting. It relies on the primary health care system and takes advantage of a simple case definition to find suspected cases allowing for an initial diagnosis by non-medical personnel [11]. This allows for the use of a wide network of community volunteers searching for cases and covering remote areas where Guinea worm is often found and where it is more difficult for more formal health workers to reach [11].

Surveillance activities are primarily in humans, although the focus is dependent on the epidemiology of the disease; recent increases in identification of cases in animals means surveillance has been expanded in some countries to include dogs [14,16] and surveillance strategies in wild animals are under consideration.

If the programme does not detect any indigenous cases of Guinea worm for a year it can move into a three year, pre-certification intensified surveillance stage [27]. This involves continued active and passive surveillance strategies. If no further cases are identified during this time period, the country can be certified as free of transmission, however surveillance needs to be maintained until global eradication is declared [28,29].

In contrast, the verification of the elimination of onchocerciasis aims to interrupt transmission but not necessarily identify all cases [30]. The onchocerciasis surveillance strategy focuses on active surveillance and primarily relies on an entomological component, monitoring infectivity rates of black flies and an epidemiological component, tracking exposure to infection in humans, through sentinel surveillance of villages and breeding sites. The primary indicator for onchocerciasis is the detection of *O*. *volvulus* DNA through O-150 PCR (poolscreen) in blackflies. Table 2 outlines the indicator in more detail. Serological indicators are also recommended as a complement to the entomological indicator, especially where collection of the required number of flies is difficult to achieve [31].

Following cessation of mass drug administration (MDA), current recommendations outline the need to reassess the entomological indicator, 3–5 years after stopping treatment [31], a timeframe by which models suggest resurgence is likely to be detected [32]. At this stage, conditional use of serology is recommended only when the entomological threshold is equal to or close to being achieved [31]. Countries are encouraged to set up National Onchocerciasis Elimination Committees (NOEC) to provide external review of programme data and provide advice to the Ministry of Health on how to achieve the elimination of onchocerciasis targets [31,33].

Finally, there is the validation of trachoma as a public health problem that does not aim to interrupt transmission and infection and morbidity may occur but at levels that are not deemed to be a problem at the level of the population [24,34]. Unlike Guinea worm, there is no imperative to quickly identify cases as for progression of the disease and significant morbidity, an individual would require multiple infections over time. If the programme is able to ensure transmission of *Ct* is kept low, potentially in a state of equilibrium, then individuals should no longer be exposed to the number of infections required to result in significant morbidity [35].

The trachoma surveillance strategy is based on active and passive components, which involve monitoring clinical indicators in humans [36]. Active trachoma is primarily monitored through the identification of TF; for diagnosis, the eyelid needs to be everted and assessed by a trained grader. In comparison TT is easier to diagnose and can be identified by lay persons with little training [37].

Trachoma surveillance and evidence for validation of elimination as a public health problem is primarily through conduct of population-based surveys, with the impact assessment to be carried out at least six months after the last round of MDA with azithromycin [36]. An evaluation unit (EU) is usually a district or administrative unit of between 100,000 and 250,000 population, with clusters being a village or an enumeration area. Passive surveillance for trachoma is related to the identification and management of incident TT cases [23].

During the pre-validation surveillance phase, the country programme must ensure that there is an effective system in place to identify and manage incident TT cases, although the specifics as to how is currently not detailed. A further population-based survey is conducted after a minimum of two years since the last survey, a timeframe chosen as it is suspected to be the earliest time-point by which resurgence will be detected at that population-level. This aims to confirm that elimination thresholds have been maintained and provide evidence for validation of elimination [23].

For all three diseases it is encouraged to include indicators as part of a country’s health management information system (HMIS) and national integrated disease and response (IDSR) system [38].

The biggest gap in current guidance for onchocerciasis and trachoma programmes, is in relation to post-elimination surveillance. This needs to continue until all countries in the region have achieved elimination status in the case of onchocerciasis and indefinitely for trachoma, however there is a paucity of information on the optimal methodology to achieve this and no formal guidelines available to guide country programmes [24,31,39].

A summary of the recommendations for Guinea worm, onchocerciasis and trachoma surveillance strategies is outlined in Table 2.

**Table 2:** Recommendations on surveillance for eradication of guinea worm and elimination of onchocerciasis and trachoma.

Consideration	Guinea worm	Onchocerciasis	Trachoma
Elimination or eradication	Eradication	Verification of elimination, interruption of transmission	Validation of elimination as a public health problem
Indicator measured	Number of cases of Guinea worm, defined as a person exhibiting a skin lesion or lesions with emergence of one or more worms, ideally that are laboratory-confirmed at CDC as *D*. *medinensis*. Each infected person is counted as a case only once during a calendar year	Entomological indicator: proportion of (parous) flies infected with infective larvae in the head. Human indicator: a measure of recent exposure through the identification of a child under 10 with antibodies to Ov-16 antigen.	Prevalence of TF–five or more follicles (0.5mm or larger) in upper central tarsal conjunctiva Prevalence of TT–one or more eyelashes in turned and touching globe of eye
Threshold targeted	Zero cases globally	It is recommended that 6,000 flies from a transmission zone are tested to provide evidence that less than 0.1% (<1/1000) parous flies have infective larvae in the head (upper bound of 95% confidence interval) or 0.5% (<1/2000) in all flies, assuming a parity rate of 50%. The black fly survey should be conducted during peak transmission season, at least six months after the last round of MDA [31]. Programmes must aim to achieve a seroprevalence of less than 0.1% in children aged under 10, with a minimum sample size of 2,000.	A TF prevalence of less than 5% in children aged 1–9 years and a TT prevalence (unknown to the health system) of less than 0.1% in the total population or 0.2% in adults aged 15 and above [36]. A TT case is known to the health system, if they have been identified by a health worker and refused surgery or have a date for surgery in place, or if they have had surgery in the past but have recurrent trichiasis.
Target group in humans	All residing in villages at risk	Children under 10 years in areas at highest risk of recrudescence	For TF: children aged 1–9 years For TT: all aged over 1
Animal surveillance	Domesticated dogs and cats	Black flies (*Simulium*)	None
Lab confirmation	Yes	Yes	No
Active surveillance	On-going door-to-door case searches in endemic or previously endemic areas or areas at risk of reintroduction. Led by community volunteers. Rumour reporting, incentivised with monetary reward for case finding.	Purposeful sampling to detect infection in black flies. Conditional use of serological indicator in children. Led by technical specialists	Population-based survey, randomly selected clusters. Led by certified graders.
Passive surveillance	Mandatory reporting through health system	No specific recommendations	System in place to identify and manage incident TT cases.
Surveillance stages	Pre-certification surveillance: 3 years from identification of last indigenous case Certification of eradication: surveillance activities to continue until global eradication	Post-treatment surveillance: 3–5 years Verification of elimination: surveillance until countries in region verified elimination	Pre-validation surveillance: 2 years Validation of elimination with post-validation elimination indefinitely

ii Comparison of active surveillance approaches

#### Community-led case surveillance

Active surveillance for Guinea worm is achieved through community-based door-to-door case finding, with a focus during peak transmission season. It is conducted in all villages that are endemic, recently endemic or at a high risk of an imported case [27]. The strategy is relatively simple and flexible in design [40] with community volunteers having an instrumental role in Guinea worm surveillance activities, leading the case searches, reporting suspected or rumour cases in a timely manner and compiling monthly reports on cases identified.

A study in Nigeria evaluated different case search approaches for Guinea worm. It utilised a mixed methods approach and highlighted the improved specificity and accuracy of case searches when utilising community reporting at farmers markets over government-led case searches [41,42]. The study highlighted the need to understand local experiences, community structures and definitions of a disease, in order to facilitate case searches and reduce misdiagnosis. However, evidence from country evaluations conversely suggested a significant false negative rate when using a community surveillance approach, which highlights the need for continued and sustained efforts by country programmes to maintain an active and efficient surveillance system, especially in previously endemic areas [43]. It was not clear at what stage of the eradication timetable this study was conducted and therefore the value of the results in a setting where every case needs to be identified and contained promptly is questionable. In addition, the study was not an RCT and was appraised as having an unclear credibility and risk of bias.

Community-based door-to-door TT case finding has also been utilised in trachoma programmes and although this approach is expensive (compared to a passive surveillance or survey approach) and time-consuming, it has the advantage of finding the majority of TT cases and allows for immediate case management [44,45]. A community-based randomized controlled trial was conducted in Tanzania and compared the utility of community treatment assistants (CTA), facilitated with training and use of TT screening cards, to usual care provision. The authors found that the use of CTAs was a viable method to identify TT cases, and although sensitivity was only 31.2%, the number of TT cases identified was 5.6 times higher than in the standard care approach. The study also recommended further efforts to reduce the number of false positives identified [37].

The importance of extending community surveillance strategies to cover cross-border areas [46], nomadic, migratory or traveller populations has been highlighted. A study in Nigeria, utilised a targeted case search for Guinea worm cases amongst the nomadic Fulani community and highlighted the need to develop a targeted approach to identify and include nomadic groups in surveillance [47]. A study in Tanzania, evaluated an enhanced surveillance system targeting newcomers and travellers in reducing prevalence of *Ct* infection. A total of 52 communities were randomised to either receive annual MDA if warranted or annual MDA again if warranted but with enhanced surveillance to identify and treat infection amongst newcomers and travellers. There was no strong evidence to suggest a difference in the change in infection prevalence amongst the two arms [48].

There appears to be utility in using community surveillance strategies either with paid community health workers (existing in health system structure) or community volunteers. However, there are issues with the sustainability of using the latter, especially if they are offered limited incentives and where support supervision is limited, or the quality is unsatisfactory. The assimilation of the community volunteers into other health programmes such as trachoma could potentially improve motivation [43,49]. However, there is also the potential to overburden volunteers and ignore the opportunity and replacement costs for the volunteer of their unpaid time [50].

#### Rumour reporting and reward systems

To address inherent weaknesses in the Guinea worm endgame, especially under-reporting of individual cases, often rural and further from health facilities [27,51], one system employed to improve case detection has been establishment of a rumour register and a cash reward system set up for voluntary reporting of a confirmed case [11]. In some countries, the reward system has been extended to encourage the reporting of and tethering of infected dogs [14].

There are suggestions that in Chad the introduction and dissemination of the monetary reward system for identifying a case, as part of the pre-certification activities, led to the identification of the 2010 outbreak. It is unclear whether this was an example of the re-introduction of the disease or there had been low level transmission since 2000 that was not detected by the previous surveillance system in place [14]. A disadvantage of using a rumour reporting system and a large cash reward incentive is that it can result in a high number of false positives reported through the system. Investigation of a total of 528 rumours of Guinea worm reported in Nigeria in 2008, identified no true cases of Guinea worm amongst them, with the majority of cases being a boil, ulcer or sore (30%) or rheumatism or arthritis (16%) [52]. There were no trials reported assessing the effectiveness of reward systems in identifying cases. Limited evidence from the utilisation of CTAs in Tanzania, also suggests the issue of substantial false positive cases for identification of TT cases using community volunteers and this is a significant limitation [37], with expectations this would only be exacerbated by the introduction of monetary incentives for reporting of cases.

#### Sentinel surveillance

Onchocerciasis surveillance is led by specialist technicians and involves the repeat monitoring of the sentinel sites in a transmission zone, which are purposefully selected, often biased to areas with the suspected highest onchocerciasis prevalence [53]. If local knowledge of the disease is not well known or there are changing ecological factors that have not been realised, then it may miss significant areas of residual transmission [54]. No studies were identified detailing research evaluating the sensitivity or specificity of the sampling strategies.

The WHO guidance for trachoma pre-validation surveillance shifted from purposeful surveillance of a limited number of sites targeted to areas believed to be the most likely endemic or with risk factors for trachoma. In 2014, the guidance was updated, shifting away from active on-going surveillance of TF because risk factors for trachoma are not well understood at the community level and there is a lack of community data for those that are known. Ultimately it was felt that such a strategy may result in data influenced by bias and chance effect [36]. However, there may be a role for more purposeful surveillance in a post-validation setting, where resources are limited.

### Population-based survey

For the decision to stop treatment and for validation of elimination, trachoma utilises a population-based survey, with approximately 30 randomly selected clusters in each EU [36]. Efforts have been made to provide a standardised protocol and methodology for conduct of the surveys, spearheaded by the Global Trachoma Mapping Project [55]. The spatial resolution for prevalence estimates for trachoma is much lower than for Guinea worm and onchocerciasis. The population-based surveillance survey is powered to the level of the EU, usually a district. It may miss smaller foci of recrudescence that are emerging, although evidence to date suggests that sub-district re-emergence does not necessarily impact on neighbouring sub-districts [36]. Further evidence is required to understand the implications of community and sub-district persistent infection or recrudescence and methodologies to detect it, especially post-validation.

TT prevalence estimates from the population-based surveys are also reported but the survey is not powered to determine if the TT thresholds have been realised [55]. To do this with the required confidence would require a survey with a larger sample size, such as outlined in a TT only survey [56], although there is still a risk that such a strategy would result in a prevalence estimate with confidence intervals inclusive of the target threshold.

In general, trachoma surveys exclude urban areas from their sampling frame. However, with increasing urbanisation leading to an exacerbation of social and sanitation problems, known to be a risk factor for trachoma, combined with an influx of people from more rural areas who may introduce trachoma, this may lead to unidentified transmission pockets. A cross-sectional survey was conducted in an urban community in Gambia and although TF prevalence was below the elimination thresholds there were communities identified with a TF prevalence of over 5% and TT remains a public health problem (over 0.1%), with the authors concluding it would be prudent to include similar urban or peri-urban areas in surveillance activities [57].

A comparison of the various active surveillance strategies are summarised in Table 3.

**Table 3:** Summary of studies evaluating evidence of surveillance strategies and implications for trachoma surveillance.

Surveillance strategy	Summary current evidence									Implications for trachoma surveillance
	GUINEA WORM			ONCHOCHERCIASIS			TRACHOMA			
	Summary of evidence from literature review	Strength of published evidence	Refs	Summary of evidence from literature review	Strength of published evidence	Refs	Summary of evidence from literature review	Strength of published evidence	Refs	
Community-led surveillance	• Improved specificity and accuracy of case searches over government-led case searches in Nigeria. • Substantial false-negative reporting rate a concern • Need for a targeted approach to identify and include nomadic groups in surveillance	Experimental evidence has unclear credibility and risk of bias	[42]; [41]; [47]	None	N/A		• Use of community door-to door case searches are used in some programme settings and found to identify more cases than through other approaches RCT conducted in Tanzania found potential utility of community assistants facilitated with training and TT screening cards to identify TT cases, but issues of sensitivity and specificity • A RCT focusing on enhanced surveillance and treatment of travellers into communities found no evidence of impact on prevalence of *Ct* infection	Observational evidence from country experiences Experimental evidence credible and low risk of bias	[44,45] [37] [48]	There is potential use for trained and facilitated community volunteers in identifying TT cases in communities. However, the optimal case search strategy is still to be determined in the endgame, when the distribution of cases is likely more sparse and rural
Rumour reporting and reward systems	Suggestions this was able to identify previously unknown foci of infections as in Chad	Limited published evidence on its effectiveness, primarily observational studies.	[14,58]	No evidence found	No experimental studies identified.		No evidence found	No experimental studies identified.		Potential utility to use reward systems for identification of TT cases. However, measures would need to be evaluated (using an experimental or quasi-experimental approach) to determine the cost-effectiveness of the system for TT and to find a balance to counter the lack of specificity in the system
Sentinel surveillance	No evidence found	No experimental studies identified		Key recommendation in the WHO elimination guidelines, biased to areas of suspected high onchocerciasis prevalence. Potential to miss significant areas of residual transmission. No studies identified detailing research evaluating the sensitivity and specificity of the sampling strategies	No experimental studies identified		WHO guidance shifted from purposeful surveillance of select sites based on a lack of community information and knowledge as to risk factors and trachoma endemicity. Potential to introduce too much bias	No experimental studies identified		A more targeted surveillance approach using sentinel surveillance could be a useful strategy in a post-validation scenario where there are limited resources. However, more evidence would be required on (causal) associations related to community risk factors and trachoma infection or improved methodologies to identify areas at risk of recrudescence
Population-based survey	No evidence found but surveys for rare events require large sample sizes and are not designed to identify all cases as required for eradication efforts	No experimental studies comparing approaches identified		No evidence found	No experimental studies comparing approaches identified		To determine stopping decision and for evidence of validation of elimination. Potentially will miss smaller foci (smaller than an administrative unit of 100-250k population) of infection. Require larger sample size for TT prevalence estimates. Exclusion of urban setting	No experimental studies comparing approaches identified		Will remain a key pre-validation surveillance activity. More evidence required as to the optimal timing of repeat surveys to identify recrudescence (pre-validation)

iii Diagnostics

This review focused on the opportunities and limitations of using infection and serological testing for surveillance, as reported in the literature. Specific papers evaluating different diagnostic tests were outside the scope of this review.

#### Test for infection

Both Guinea worm and onchocerciasis surveillance include a test for infection within their surveillance strategy. For Guinea worm this has been introduced latterly as numbers of Guinea worm cases have reduced and worms emerge in isolation. Retrieved Guinea worms are subject to molecular testing and evaluation of the morphology of the worm [14,28] to confirm they are *D*. *medinensis* and not confused with other worms e.g *O*. *volvulus* [59,60].

The primary indicator for onchocerciasis, relating to infectivity in black flies, is measured through PCR testing for *O*. *volvulus*. This has the advantage that it is a very specific test and can distinguish between the human infection and *O*. *ochengi* that can be found in cattle [31]. A key disadvantage is that PCR for detection of *O*. *volvulus* in flies is not readily available, with only a few laboratories having the technical capacity to conduct the test, especially in onchocerciasis endemic countries [54]. There are also significant costs associated with black fly DNA testing, although this is mitigated in part, through the use of pool screening [31].

For trachoma where a low pathogen load is unlikely to lead to transmission of infection and where a low level of on-going transmission is in line with elimination as a public health problem, the specificity of the surveillance system has been deemed critical [12]. However, it is known there are issues with the specificity of the clinical indicator in low prevalence settings with a discordance between TF and ocular *Ct* infection [61–64] and subjectivity in the inter-grader agreement for borderline TF cases [36]. Current evidence from an elimination setting where TF is very low, at only 1 or 2% and well below the 5% elimination threshold, has shown *Ct* infection to be very low or non-detectable [65,66]. Therefore, in this context, current evidence suggests direct evaluation of ocular infection does not make a difference to programmatic decision-making or validation of elimination but may delay results and a timely response. However, current guidelines would denote an EU where the TF prevalence estimate rose from 4.9% to 5.1% as having fit the criteria for recrudescence, even if the lower bound 95% confidence interval of the estimate dipped below 5.0%. As TF can also be a symptom of other infections or there can be a delay in TF disappearing even when *Ct* infection has been cleared, the evaluation of *Ct* infection, especially where there are prevalence estimates close to the threshold cut-off can be used to clarify if there is true recrudescence of trachoma that would require programmatic intervention.

#### Serological test

Onchocerciasis is the only one of the three diseases that has a specific serological target for surveillance. In low prevalence settings or post-treatment, the use of serology (anti Ov-16 antibodies) is deemed more sensitive [31,67] for monitoring transmission than the use of microscopy to identify microfilariae in skin snips, which historically have been the mainstay of onchocerciasis monitoring in humans. Rapid antibody tests have also been shown to be preferable to the community, with an evaluation in Senegal highlighting improved participation for the antibody test, with 99.7% uptake as compared to 32.7% for the skin snip biopsy [68].

A serologic target can not distinguish between current infection and past exposure. However, for onchocerciasis, it is expected that children aged 10 and under will be infection naïve if the programme has been successful in suppressing transmission (over a 10 year period or life of adult worm) [69]. The assumption is that all infections lead to seroconversion, however a significant proportion of individuals (15–25%) have been found to not illicit an anti Ov-16 immune response [70]. Although a low target threshold may help to counteract this loss in sensitivity, any target chosen must be achievable. The target for onchocerciasis programmes (<0.1% seroprevalence in children under 10) would require a diagnostic test with consistently greater than 99.9% specificity, which is not possible with current platforms available to country programmes [67]. For this reason, the target is currently under review.

Finally, the methodology used to determine a seropositive individual makes a difference to the overall seroprevalence estimates in a population [71], yet the onchocerciasis guidelines do not specify the test required (rapid test or ELISA) to determine an Ov16 seropositive individual nor the methodology to determine seropositive thresholds [31]. In trachoma endemic areas serology when combined with age can be a useful indicator to help in understanding historical transmission dynamics and potentially earmark areas at risk of recrudescence, which can be monitored more closely as part of a post-validation surveillance strategy [65,66,72–76]. The main disadvantage is that the currently favoured antibody of interest (anti-Pgp3) can not distinguish between urogenital or ocular chlamydia and there are still gaps in the understanding of an individual’s immunological response [77]. To date, clinical indicators continue to be the only diagnostic recommended for programmatic decision-making.

A summary of the considerations for laboratory confirmation of trachoma infection and exposure is highlighted in Table 4.

**Table 4:** Summary of studies evaluating use of laboratory confirmation for surveillance.

Surveillance strategy	Summary of current evidence (elimination setting)						Implications for trachoma surveillance
	GUINEA WORM		ONCHOCHERCIASIS		TRACHOMA		
	Summary of recommendation	Lessons learnt	Summary of recommendation	Lessons learnt	Summary of recommendation	Lessons learnt	
Test for infection	Molecular testing and morphological evaluation of the worms.	In the endgame it is important to confirm all cases utilising a specific test [12]	Pool PCR to test for *O*. *volvulus* in black flies [31]	Improved specificity of diagnosis (in particular to distinguish from *O*. *ochengi*) [31]. Expensive test to conduct although costs partly mitigated through pooling of samples. Through-put limited by availability of platforms for test, impacting on capacity for timely decision-making.	Not recommended at present	Disparity between TF and *Ct* in low prevalence settings. However, in general an EU with a TF prevalence in children aged 1–9 years of less than 5% also equates to a low prevalence of *Ct* infection [65,66,76]	Cost and capacity issues likely hinder the roll out of a PCR tests at scale as part of programmatic decision-making. However, a more targeted use for confirmation of the prevalence of infection in EUs on the border of the TF elimination threshold or where there has been suspected recrudescence of infection, would likely be useful.
Serological test	None utilised	Not applicable	Recommended as a complement to entomological surveillance. Specific serologic target in children (anti Ov-16 antibodies) [31]	Important achievable target thresholds set, taking into account sensitivity and specificity of available tests [67]. Long half-life of antibodies and wide target age range can lead to misinterpretation of findings. May be beneficial to have differing antibody thresholds in line with baseline epidemiological thresholds.	Not recommended at present	Evaluation of serological indicators combined with age appear to be useful to determine historical transmission dynamics [65,72]	Further research required to determine the potential utility of serological indicators (potentially as part of an adaptive sampling approach) to identify areas at risk of recrudescence.

TF: Trachoma inflammation—follicular; *Ct*: *Chlamydia trachomatous*; EU: Evaluation unit; *Ov*: *Onchocerca volvulus*

## Discussion

Due to the clinical progression of trachoma, with the active infectious form of trachoma and TT, the progression of the disease, adds complexity to trachoma, with two related but morphologically different presentations, which require alternative surveillance strategies. Trachoma pre-validation surveillance has three key functions, firstly the monitoring of trachoma to determine if elimination thresholds have been achieved, the identification of any recrudescence in a timely manner and finally the identification and management of incident TT cases [36]. There are a number of lessons learnt from the Guinea worm surveillance system that are particularly compatible for TT surveillance while the onchocerciasis surveillance strategy can provide insights for surveillance of the active infectious form of trachoma.

For Guinea worm where the target is eradication, it is important to have a sensitive system that is able to detect cases in a timely manner, a goal facilitated by rumour reporting and the reward scheme for reporting of a confirmed case of Guinea worm and the exhaustive door-to-door case search using community volunteers. Although trachoma is not an eradication programme it would be preferable if a programme was able to identify and manage all cases of trichiasis, as the condition is painful and can lead to sight loss if left untreated. Currently TT elimination thresholds are primarily monitored through a survey methodology, which can lead to imprecise prevalence estimates. In addition, passive surveillance systems have been shown to under-estimate TT cases [45]. Ghana and other countries including Morocco [45,78] have successfully used door-to-door case searches to identify TT cases and more accurately determine if TT elimination thresholds have been met. However, due to the cost and logistical implications, it is necessary to be strategic as to when and where such strategies are employed. The utility of the reward system, as used in Guinea worm surveillance, to identify trichiasis cases, which can be diagnosed by non-medical personnel, may be an interesting application requiring further exploration. However, the use of community volunteers to identify TT cases has already been shown to result in a high proportion of false positive cases being referred [37] and there is a risk that implementing such a reward system would exacerbate this problem. As there is little objective evidence as to the effectiveness of the reward system in identifying confirmed cases of Guinea worm and previously unknown foci of transmission, a suitably designed RCT, with appropriate formative research understanding motivational factors for case finding, would be beneficial to evaluate the evidence of the effectiveness of this approach for TT case finding.

The specificity of a trachoma surveillance system is critical in order to ensure elimination targets are effectively determined and interventions are not prolonged, wasting limited resources [12]. The onchocerciasis surveillance system has had a key focus on introducing appropriate diagnostic tests to measure transmission parameters, primarily infection in black flies and serological markers in humans. However, the tests introduced have been complex, with restricted global capacity to implement, which can unduly delay programme decision making. The target thresholds have not been based on sound evidence and the serological targets are practically unattainable with current diagnostic tests [67].

Detection of ocular *Ct* infection through PCR in trachoma has been shown to be useful in understanding the true infection rates [61,62,64,65] but is expensive, although pooling of samples for analysis can mitigate the cost to an extent [79–81]; so will likely be better targeted as a resource, for instance to determine true levels of infection in EUs with a TF prevalence close to the elimination threshold.

The current serological threshold for verification of elimination of onchocerciasis is <0.1% in children under 10 years. Although a direct comparison can not be made, in part because the aim for trachoma is not interruption of transmission, evidence from modelling suggests a suitable serological threshold for trachoma that equates to a TF prevalence of <5% in children aged 1–9 years, would be a seroprevalence (anti-Pgp3 antibodies) of 7.3% (95%CI: 6.5–8.3) [82]. The use of a fixed serological cut-off for a measure of exposure, that encompasses a wide age range can have issues, as historical transmission and the long antibody half-lives, can lead to a misinterpretation of findings. Further the cross-reactivity of the antibodies to multiple antigens, whether it be other filaria or in the case of trachoma, urogenital chlamydia, means that epidemiological context must be taken into account when determining serological cut-offs. There is potentially greater utility in using serology as an indicator of recent transmission dynamics [83] and for aiding the identification of areas at potential increased risk of recrudescence [65,71,72]. Serology is also a useful indicator as it can easily be integrated with the monitoring of other infectious diseases [12,84]. However, there are a number of factors that need to be considered before any recommendations to include infection or serological marker within trachoma surveillance, including a standardised methodology for determining a seropositive individual (76) and a network of laboratories that can conduct serological tests with necessary proficiency or a point of care or rapid test that would negate it.

Trachoma currently utilises a population-based survey methodology for validation of elimination. Purposeful selection of sentinel sites as a surveillance strategy is no longer recommended pre-validation [36]. However, there is potential utility of a more targeted surveillance approach in a post-validation setting, where population-based survey approaches are not warranted. Such an approach could focus on surveillance of communities at increased risk of recrudescence. Further work is required to understand the risk factors and assist in defining targets for surveillance, however, the use of infection and serology data shows potential promise as a strategy [65], potentially as part of an adaptive sampling approach.

It would have been useful to frame this paper based on the elimination dossiers of the ten countries that have been validated as having eliminated trachoma as a public health problem, however, very few were publicly available. The literature search highlighted very few published papers evaluating the integration of surveillance systems into more established care pathways, something that is likely to be key for sustainable surveillance in the endgame and a topic that this review would have benefited from further discussion on. The paper also only reviews lessons learnt from two NTDs and it could be useful to extend such a review to include other pertinent diseases, for example lymphatic filariasis, that also has a surveillance system that requires monitoring of infection and chronic morbidity. Finally, the literature review methodology utilised for this paper was not intended to be that of a systematic review, however, the authors still aimed to ensure a rigorous methodology, with clear inclusion and exclusion criteria and an assessment of the credibility and risk of bias amongst papers included in order to understand the strength of evidence available. It is felt that this approach has highlighted some interesting lessons learnt, as well as inform current evidence gaps that would be useful for further research.

## Conclusion

The experiences of both the Guinea worm and onchocerciasis surveillance strategies have very useful lessons for trachoma surveillance, pre- and post-validation. The use of a monetary reward for identification of TT cases and further exploration into the use of infection and serological indicators particularly in a post-validation setting to assist in identifying recrudescence would be of particular relevance. The next step would be a real-world evaluation of their relative applicability for trachoma surveillance.

## Supporting information

S1 Table Exhaustive list of search terms used. (DOCX)

S2 Table List of papers eligible for inclusion. (DOCX)
