# Supplementary material for: Lessons learned for surveillance strategies for trachoma elimination as a public health problem, from the evaluation of approaches utilised by Guinea worm and onchocerciasis programmes: A literature review
Source: PLoS Negl Trop Dis. 2021 Jan 28;15(1):e0009082. doi: 10.1371/journal.pntd.0009082 (PMC7872237; doi:10.1371/journal.pntd.0009082)
Supplement: S1 Table — (DOCX) [file pntd.0009082.s001.docx]

**S1 Table: Exhaustive list of search terms used**

| Search terms used in PUBMED | Search terms used in Google Scholar |
| --- | --- |
| guinea AND worm AND surveillance | guinea worm surveillance |
| dracunculiasis AND surveillance | onchocerciasis surveillance |
| guinea AND worm AND eradication | trachoma surveillance |
| dracunculiasis AND eradication | neglected tropical disease surveillance |
| onchocerciasis AND surveillance |  |
| river AND blindness AND surveillance |  |
| onchocerciasis AND elimination |  |
| onchocerciasis AND verification |  |
| trachoma* AND surveillance |  |
| trachoma* AND elimination |  |
| trachoma* AND validation |  |
| neglected AND tropical AND disease AND surveillance |  |
