# Supplementary material for: Lessons learned for surveillance strategies for trachoma elimination as a public health problem, from the evaluation of approaches utilised by Guinea worm and onchocerciasis programmes: A literature review
Source: PLoS Negl Trop Dis. 2021 Jan 28;15(1):e0009082. doi: 10.1371/journal.pntd.0009082 (PMC7872237; doi:10.1371/journal.pntd.0009082)
Supplement: S2 Table — (DOCX) [file pntd.0009082.s002.docx]

S2 Table: List of papers eligible for inclusion

|  | **Title** | **Date** | **First author** | **Type** | **Critical appraisal** | **Reference** |
| --- | --- | --- | --- | --- | --- | --- |
| 1 | Strategies for dracunculiasis eradication | 1991 | Hopkins | Description of country experiences | N/A | [1] |
| 2 | The peculiar epidemiology of dracunculiasis in Chad | 2014 | Eberhard | Description of country experiences | N/A | [2] |
| 3 | Elimination of Guinea worm disease in Ethiopia; current status of the disease, eradication dtrategies and challenges to the end game | 2017 | Beyene | Description of country experiences | N/A | [3] |
| 4 | Report on the Meeting on post-endemic surveillance for blinding trachoma; WHO, Geneva, 4–5 November 2008 | 2008 | WHO | Guidelines | N/A | [4] |
| 5 | Validation of elimination of trachoma as a public health problem | 2016 | WHO | Guidelines | N/A | [5] |
| 6 | Criteria for certification of dracunculiasis elimination | 1996 | Tayeh | Guidelines | N/A | [6] |
| 7 | Certification of elimination of human onchocerciasis: criteria and procedures | 2000 | WHO | Guidelines | N/A | [7] |
| 8 | Guidelines for stopping mass drug administration and verifying elimination of human onchocerciasis | 2016 | WHO | Guidelines | N/A | [8] |
| 9 | Modelling the elimination of river blindness using long-term epidemiological and programmatic data from Mali and Senegal | 2017 | Walker | Modelling | N/A | [9] |
| 10 | The role of national committees in eliminating onchocerciasis | 2018 | Griswold | Description of country experiences | N/A | [10] |
| 11 | Technical consultation on trachoma surveillance; Task Force for Global Health, Decatur, USA, September 11−12, 2014 | 2014 | WHO | Guidelines | N/A | [11] |
| 12 | Assessment of a novel approach to identify trichiasis cases using community treatment assistants in Tanzania | 2015 | Greene | Research evaluation | Credible and low risk bias | [12] |
| 13 | Technical guidelines for Integrated Disease Surveillance and Response in the African Region, second edition | 2010 | WHO | Guidelines | N/A | [13] |
| 14 | Evaluation of community-based surveillance for Guinea worm, South Sudan, 2006 | 2006 | Lado | Research evaluation | Unclear credibility and risk of bias | [14] |
| 15 | Learning from local knowledge to improve disease surveillance: perceptions of the guinea worm illness experience | 1992 | Brieger | Research evaluation | Unclear credibility and risk of bias | [15] |
| 16 | The Yoruba farm market as a communication channel in guinea worm disease surveillance | 1996 | Brieger | Research evaluation | Unclear credibility and risk of bias | [16] |
| 17 | Monitoring and evaluation of Guinea worm eradication | 1998 | Périès | Description of country experiences |  | [17] |
| 18 | Use of validated community-based trachoma trichiasis (TT) case finders to measure the total backlog and detect when elimination threshold is achieved: a TT methodology paper | 2017 | Karimurio | Research evaluation | Credible and low risk bias | [18] |
| 19 | Dossier documenting elimination of trachoma as a public health problem, Ghana | 2018 | GHS | Description of country experiences | N/A | [19] |
| 20 | Cross-border issues: an important component of onchocerciasis elimination programmes | 2018 | Bush | Description of country experiences | N/A | [20] |
| 21 | Ethnic diversity and disease surveillance: Guinea worm among the Fulani in a predominantly Yoruba district of Nigeria | 1997 | Brieger | Research evaluation | Risk of bias | [21] |
| 22 | Treating village newcomers and travelers for trachoma: Results from ASANTE cluster randomized trial | 2017 | West | Research evaluation | Credible and low risk bias | [22] |
| 23 | Assessment of the community-based surveillance system in Ghana and its role in dracunculiasis eradication | 2003 | Unknown | Description of country experiences | N/A | [23] |
| 24 | Evaluation of the dracunculiasis surveillance system in four districts in Ghana | 2005 | WHO | Research evaluation | Unclear credibility and risk of bias | [24] |
| 25 | Guinea worm wrap-up #188 |  | WHO | Description of country experiences | N/A | [25] |
| 26 | Update on the current status of onchocerciasis in Côte d’Ivoire following 40 years of intervention: Progress and challenges | 2018 | Koudou | Description of country experiences | N/A | [26] |
| 27 | Transitioning from river blindness control to elimination: steps toward stopping treatment | 2018 | Cantey | Review of applicability of current guidelines |  | [27] |
| 28 | The Global Trachoma Mapping Project: Methodology of a 34-country population-based study | 2015 | Solomon | Description of country experiences | N/A | [28] |
| 29 | Design and validation of a trachomatous trichiasis-only survey | 2018 | WHO | Guidelines | N/A | [29] |
| 30 | Follicular trachoma and trichiasis prevalence in an urban community in The Gambia, West Africa: is there a need to include urban areas in national trachoma surveillance? | 2013 | Quicke | Research evaluation | Credible and low risk bias | [30] |
| 31 | Renewed transmission of dracunculiasis - Chad, 2010 | 2011 | Unknown | Description of country experiences | N/A | [31] |
| 32 | Serological and PCR-based markers of ocular *Chlamydia trachomatis* transmission in northern Ghana after elimination of trachoma as a public health problem | 2018 | Senyonjo | Research evaluation | Credible and low risk bias | [32] |
| 33 | Surveillance surveys for re-emergent trachoma in formerly endemic districts in Nepal from 2 to 10 Years after mass drug administration cessation | 2017 | West | Research evaluation | Credible and low risk bias | [33] |
| 34 | Rethinking the serological threshold for onchocerciasis elimination | 2018 | Gass | Review of applicability of current guidelines | N/A | [34] |
| 35 | Feasibility of utilizing the SD BIOLINE Onchocerciasis IgG4 rapid test in onchocerciasis surveillance in Senegal | 2017 | Dieye | Research evaluation | Credible and low risk bias | [35] |
| 36 | Serology reflects a decline in the prevalence of trachoma in two regions of The Gambia | 2017 | Migchelsen | Research evaluation | Credible and low risk bias | [36] |
| 37 | Serology for trachoma surveillance after cessation of mass drug administration | 2015 | Martin | Research evaluation | Credible and low risk bias | [37] |
| 38 | Prevalence of *Chlamydia trachomatis*-specific antibodies before and after mass drug administration for trachoma in community-wide surveys of four communities in Nepal | 2018 | Gwyn | Research evaluation | Credible and low risk bias | [38] |
| 39 | Community-level chlamydial serology for assessing trachoma elimination in trachoma-endemic Niger | 2019 | Kim | Research evaluation | Credible and low risk bias | [39] |
| 40 | Can We Use antibodies to *Chlamydia trachomatis* as a surveillance tool for national trachoma control programs? Results from a district survey | 2016 | West | Research evaluation | Credible and low risk bias | [40] |
| 41 | The utility of serology for elimination surveillance of trachoma | 2018 | Pinsent | Modelling | N/A | [41] |

**References**

1. Hopkins DR, Ruiz-Tiben E. Strategies for dracunculiasis eradication. Bull World Hlth Org. 1991;69(5):533-40.

2. Eberhard ML, Ruiz-Tiben E, Hopkins DR, Farrell C, Toe F, Weiss A, et al. The peculiar epidemiology of dracunculiasis in Chad. Am J Trop Med & Hlth. 2014;90(1):61-70.

3. Beyene HB, Bekele A, Shifara A, Ebstie YA, Desalegn Z, Kebede Z, et al. Elimination of Guinea Worm Disease in Ethiopia; Current Status of the Disease's, Eradication Strategies and Challenges to the End Game. Ethiop Med J. 2017;55(Suppl 1):15-31.

4. WHO. Report on the meeting on post-endemic surveillance for blinding trachoma. Geneva: WHO; 2008.

5. WHO. Validation of elimination of trachoma as a public health problem. Geneva: WHO; 2016.

6. Tayeh A, unit FC. Criteria for certification of dracunculiasis elimination. Geneva: WHO; 1996.

7. WHO. Certification of eliminationof human onchocerciasis: criteria and procedures, Geneva, 28-29 September 2000 (doc ref. WHO/CDS/CPE/CEE/2001.18a). Geneva, Swtizerland: WHO; 2000.

8. WHO. Guidelines for stopping mass drug administration and verifying elimination of human onchocerciasis. Criteria and procedures. Geneva: WHO; 2016.

9. Walker M, Stolk WA, Dixon MA, Bottomley C, Diawara L, Traoré MO, et al. Modelling the elimination of river blindness using long-term epidemiological and programmatic data from Mali and Senegal. Epidemics. 2017;18:4-15.

10. Griswold E, Unnasch T, Eberhard M, Nwoke BEB, Morales Z, Muheki Tukahebwa E, et al. The role of national committees in eliminating onchocerciasis. Int Hlth. 2018;10(suppl_1):i60-i70.

11. WHO. Technical consultation on trachoma surveillance, September 11-14, 2014. Decatur: Task Force for Global Health; 2015.

12. Greene GS, West SK, Mkocha H, Munoz B, Merbs SL. Assessment of a Novel Approach to Identify Trichiasis Cases Using Community Treatment Assistants in Tanzania. PLoS NTD. 2015;9(12):e0004270.

13. WHO, CDC. Technical guidelines for integrated disease surveillance and response for the african region, 2nd edition. 2010.

14. Lado M, Mackoy S, Steve B, Rumunu J. Evaluation of community-based surveillance for Guinea worm, South Sudan, 2006. South Med J. 2002;5(3).

15. Brieger WR, Kendall C. Learning from local knowledge to improve disease surveillance: perceptions of the guinea worm illness experience. Hlth Educ Res. 1992;7(4):471-85.

16. Brieger WR, Kendall C. The Yoruba farm market as a communication channel in guinea worm disease surveillance. Soc Sci Med (1982). 1996;42(2):233-43.

17. Périès H, Rooy Cd, Nwe YY. Monitoring and evaluation of Guinea Worm Eradication. Eval Program Plan. 1998;21(4):393-408.

18. Karimurio J, Rono H, Njomo D, Sironka J, Kareko C, Gichangi M, et al. Use of validated community-based trachoma trichiasis (TT) case finders to measure the total backlog and detect when elimination threshold is achieved: a TT methodology paper. Pan Afr Med J. 2017;27:84-.

19. GHS GHS-. Dossier documenting elimination of trachoma as a public health problem. Accra: GHS; 2018.

20. Bush S, Sodahlon Y, Downs P, Mackenzie CD. Cross-border issues: an important component of onchocerciasis elimination programmes. Int Hlth. 2018;10(suppl_1):i54-i9.

21. Brieger WR, Oke GA, Otusanya S, Adesope A, Tijanu J, Banjoko M. Ethnic diversity and disease surveillance: Guinea worm among the Fulani in a predominantly Yoruba district of Nigeria. TM & IH. 1997;2(1):99-103.

22. West SK, Munoz B, Mkocha H, Dize L, Gaydos CA, Swenor B, et al. Treating village newcomers and travelers for trachoma: Results from ASANTE cluster randomized trial. PLoS One. 2017;12(6):e0178595.

23. Unknown. Assessment of the Community-Based Surveillance System in Ghana and its role in dracunculiasis eradication. WER. 2003;78(37):321-3.

24. Evaluation of the dracunculiasis surveillance system in 4 districts in Ghana. WER. 2005;80(32):270-6.

25. WHO. GUINEA WORM WRAP-UP #188 Geneva, Switzerland: WHO; 2008.

26. Koudou BG, Kouakou M-M, Ouattara AF, Yeo S, Brika P, Meite A, et al. Update on the current status of onchocerciasis in Côte d’Ivoire following 40 years of intervention: Progress and challenges. PLoS NTD. 2018;12(10):e0006897.

27. Cantey PT, Roy SL, Boakye D, Mwingira U, Ottesen EA, Hopkins AD, et al. Transitioning from river blindness control to elimination: steps toward stopping treatment. Int Hlth. 2018;10(suppl_1):i7-i13.

28. Solomon AW, Pavluck AL, Courtright P, Aboe A, Adamu L, Alemayehu W, et al. The Global Trachoma Mapping Project: Methodology of a 34-Country Population-Based Study. Ophthalmic Epi. 2015;22(3):214-25.

29. WHO Strategic and Technical Advisory Group on Neglected Tropical Diseases. Design and validation of a trachomatous trichiasis-only survey (WHO/HTM/NTD/PCT/2017.08). . Geneva: WHO; 2018.

30. Quicke E, Sillah A, Harding-Esch EM, Last A, Joof H, Makalo P, et al. Follicular trachoma and trichiasis prevalence in an urban community in The Gambia, West Africa: is there a need to include urban areas in national trachoma surveillance? TM & IH. 2013;18(11):1344-52.

31. Renewed transmission of dracunculiasis--Chad, 2010. MMWR Morb Mortal Wkly Rep. 2011;60(22):744-8.

32. Senyonjo LG, Debrah O, Martin DL, Asante-Poku A, Migchelsen SJ, Gwyn S, et al. Serological and PCR-based markers of ocular *Chlamydia trachomatis* transmission in northern Ghana after elimination of trachoma as a public health problem. PLoS NTD. 2018;12(12):e0007027-e.

33. West SK, Zambrano AI, Sharma S, Mishra SK, Munoz BE, Dize L, et al. Surveillance Surveys for Reemergent Trachoma in Formerly Endemic Districts in Nepal From 2 to 10 Years After Mass Drug Administration Cessation. JAMA Ophthalmol. 2017;135(11):1141-6.

34. Gass KM. Rethinking the serological threshold for onchocerciasis elimination. PLoS NTD. 2018;12(3):e0006249.

35. Dieye Y, Storey HL, Barrett KL, Gerth-Guyette E, Di Giorgio L, Golden A, et al. Feasibility of utilizing the SD BIOLINE Onchocerciasis IgG4 rapid test in onchocerciasis surveillance in Senegal. PLoS NTD. 2017;11(10):e0005884.

36. Migchelsen SJ, Sepulveda N, Martin DL, Cooley G, Gwyn S, Pickering H, et al. Serology reflects a decline in the prevalence of trachoma in two regions of The Gambia. Sci Rep. 2017;7(1):15040.

37. Martin DL, Bid R, Sandi F, Goodhew EB, Massae PA, Lasway A, et al. Serology for trachoma surveillance after cessation of mass drug administration. PLoS NTD. 2015;9(2):e0003555.

38. Gwyn SE, Xiang L, Kandel RP, Dean D, Gambhir M, Martin DL. Prevalence of *Chlamydia trachomatis*-Specific Antibodies before and after Mass Drug Administration for Trachoma in Community-Wide Surveys of Four Communities in Nepal. Am J Trop Med & Hyg. 2018;98(1):216-20.

39. Kim JS, Oldenburg CE, Cooley G, Amza A, Kadri B, Nassirou B, et al. Community-level chlamydial serology for assessing trachoma elimination in trachoma-endemic Niger. PLoS NTD. 2019;13(1):e0007127.

40. West SK, Munoz B, Weaver J, Mrango Z, Dize L, Gaydos C, et al. Can We Use Antibodies to Chlamydia trachomatis as a Surveillance Tool for National Trachoma Control Programs? Results from a District Survey. PLoS NTD. 2016;10(1):e0004352.

41. Pinsent A, Solomon AW, Bailey RL, Bid R, Cama A, Dean D, et al. The utility of serology for elimination surveillance of trachoma. Nat Comms. 2018;9(1):5444.
